# Supplementary material for: Complex‐centric proteome profiling by SEC‐SWATH‐MS
Source: Mol Syst Biol. 2019 Jan 14;15(1):e8438. doi: 10.15252/msb.20188438 (PMC6346213; doi:10.15252/msb.20188438)
Supplement: Supplementary file 6 — Dataset EV5 [file MSB-15-e8438-s006.zip › feature_plots_corum/739.pdf]

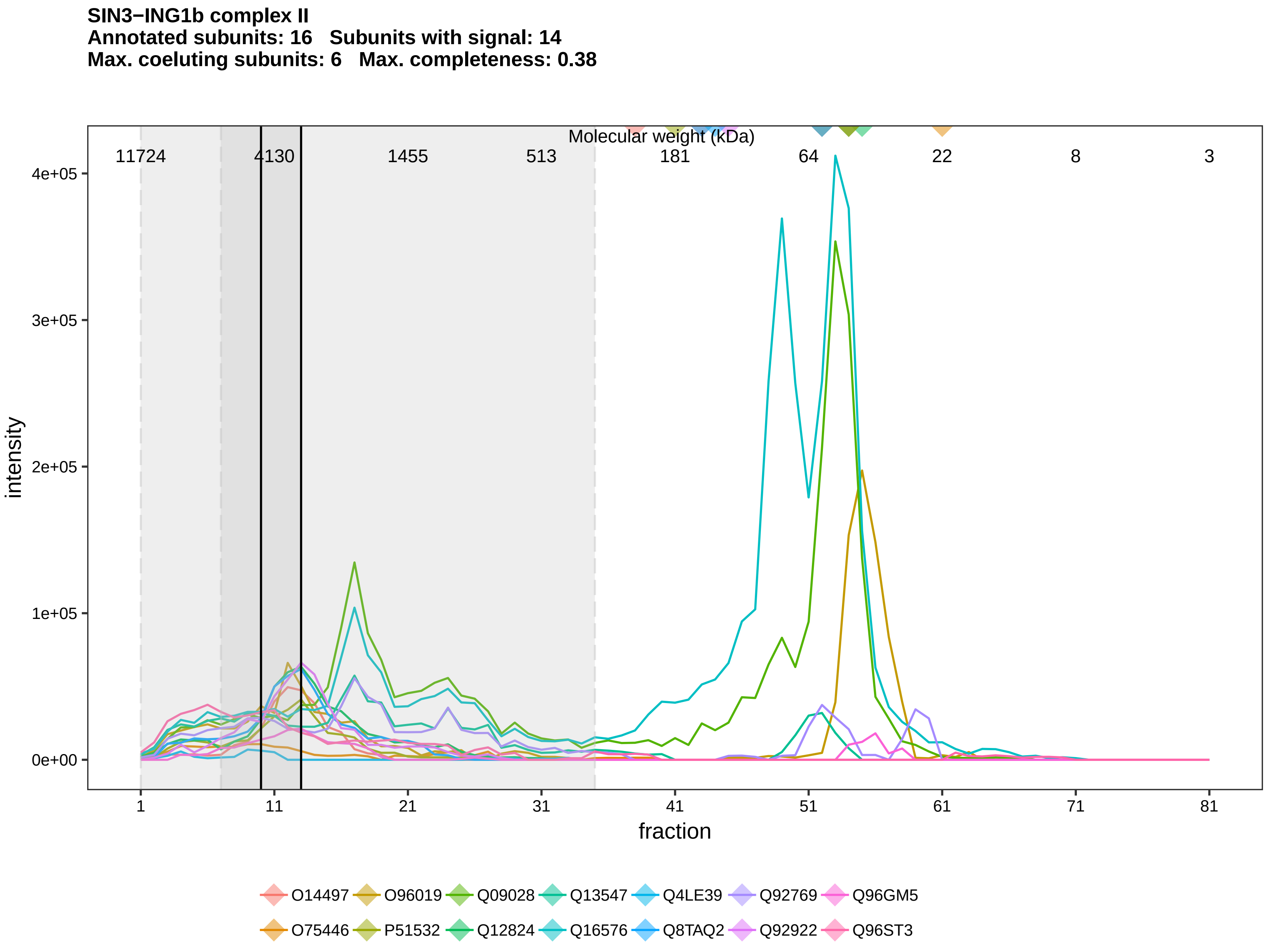

SIN3−ING1b complex II
Annotated subunits: 16 Subunits with signal: 14
Max. coeluting subunits: 6 Max. completeness: 0.38
Molecular weight (kDa)
11724
4130
1455
513
181
64
22
8
3
intensity
4e+05
3e+05
2e+05
1e+05
0e+00
fraction
1
11
21
31
41
51
61
71
81
O14497
O96019
Q09028
Q13547
Q4LE39
Q92769
Q96GM5
O75446
P51532
Q12824
Q16576
Q8TAQ2
Q92922
Q96ST3
